# *IBTK* Differently Modulates Gene Expression and RNA Splicing in HeLa and K562 Cells

**DOI:** 10.3390/ijms17111848

**Published:** 2016-11-07

**Authors:** Giuseppe Fiume, Annarita Scialdone, Francesca Rizzo, Maria Rosaria De Filippo, Carmelo Laudanna, Francesco Albano, Gaetanina Golino, Eleonora Vecchio, Marilena Pontoriero, Selena Mimmi, Simona Ceglia, Antonio Pisano, Enrico Iaccino, Camillo Palmieri, Sergio Paduano, Giuseppe Viglietto, Alessandro Weisz, Giuseppe Scala, Ileana Quinto

**Affiliations:** 1 Department of Experimental and Clinical Medicine, University ‘Magna Graecia’ of Catanzaro, Viale Europa (Località Germaneto), 88100 Catanzaro, Italy; annarita.scialdone@med.lu.se (A.S.); albano@unicz.it (F.A.); golino@unicz.it (G.G.); eleonoravecchio@unicz.it (E.V.); pontoriero@unicz.it (M.P.); mimmi@unicz.it (S.M.); ceglia@unicz.it (S.C.); pisano@unicz.it (A.P.); iaccino@unicz.it (E.I.); cpalmieri@unicz.it (C.P.); viglietto@unicz.it (G.V.); 2 Laboratory of Molecular Medicine and Genomics, Department of Medicine, Surgery and Dentistry ‘Schola Medica Salernitana’, University of Salerno, via S. Allende 1, 84081 Baronissi, Italy; frizzo@unisa.it (F.R.); mdefilippo@unisa.it (M.R.D.F.); laudanna@unicz.it (C.L.); aweisz@unisa.it (A.W.); 3 Department of Health Sciences, University “Magna Graecia”, 88100 Catanzaro, Italy; paduano@unicz.it

**Keywords:** Next Generation Sequencing, IBTK, Cul3-dependent E3 ligase, transcription

## Abstract

The *IBTK* gene encodes the major protein isoform IBTKα that was recently characterized as substrate receptor of Cul3-dependent E3 ligase, regulating ubiquitination coupled to proteasomal degradation of Pdcd4, an inhibitor of translation. Due to the presence of Ankyrin-BTB-RCC1 domains that mediate several protein-protein interactions, IBTKα could exert expanded regulatory roles, including interaction with transcription regulators. To verify the effects of IBTKα on gene expression, we analyzed HeLa and K562 cell transcriptomes by RNA-Sequencing before and after *IBTK* knock-down by shRNA transduction. In HeLa cells, 1285 (2.03%) of 63,128 mapped transcripts were differentially expressed in *IBTK*-shRNA-transduced cells, as compared to cells treated with control-shRNA, with 587 upregulated (45.7%) and 698 downregulated (54.3%) RNAs. In K562 cells, 1959 (3.1%) of 63128 mapped RNAs were differentially expressed in *IBTK*-shRNA-transduced cells, including 1053 upregulated (53.7%) and 906 downregulated (46.3%). Only 137 transcripts (0.22%) were commonly deregulated by *IBTK* silencing in both HeLa and K562 cells, indicating that most IBTKα effects on gene expression are cell type-specific. Based on gene ontology classification, the genes responsive to IBTK are involved in different biological processes, including in particular chromatin and nucleosomal organization, gene expression regulation, and cellular traffic and migration. In addition, *IBTK* RNA interference affected RNA maturation in both cell lines, as shown by the evidence of alternative 3′- and 5′-splicing, mutually exclusive exons, retained introns, and skipped exons. Altogether, these results indicate that *IBTK* differently modulates gene expression and RNA splicing in HeLa and K562 cells, demonstrating a novel biological role of this protein.

## 1. Introduction

The human inhibitor of Bruton’s tyrosine kinase (*IBTK*) gene (ENSG00000005700) includes 29 exons with two promoters and transcriptional start sites, and expresses three major transcripts, named *IBTK*α (ENST00000306270), *IBTK*β (ENST00000369751), and *IBTK*γ (ENST00000471036) [1,2]. The intron 26 of the *IBTK* gene encodes a pre-miR-*IBTK*3, which is cleaved by Dicer to generate 22 nucleotide-long products [3]. The three *IBTK* transcripts encode the protein isoforms IBtkα (1353 aa), IBtkβ (1196 aa), and IBtkγ (240 aa) [1]. IBtkγ was the first protein isoform to be characterized as negative regulator of Bruton tyrosine kinase (Btk) and B-cell receptor-dependent calcium flux and NF-κB activation [2,4]. IBtkα is the most expressed protein isoform that, in addition to the carboxy-terminal amino acid sequence overlapping IBtkγ, contains two Ankyrin repeats [1,5,6], three Regulators of Chromosome Condensation 1 (RCC1) motifs [7], and two Broad-Complex, Tramtrack and Bric-a-brac/POxvirus and Zinc finger (BTB/POZ) domains [8]. The presence of these protein-protein interactions domains suggests the possibility of multiple interactions of IBtkα with cellular factors.

Several members of the BTB family interact with Cul3-based SCF-like complexes, catalyzing the ubiquitination of proteins targeted for proteasomal degradation [9]. Consistently, we have recently proven that IBtkα is a component of Cul3-dependent E3 Ligase (CRL3), promoting auto-ubiquitination and ubiquitination of Pdcd4, a tumor suppressor protein acting as translation inhibitor of specific mRNAs [10,11]. In particular, the interaction of IBtkα with Pdcd4 occurred upon serum restoration in serum-starved HeLa cells, and resulted in the ubiquitination coupled to proteasomal degradation of Pdcd4, increasing the translation of specific mRNAs through counteraction of Pdcd4 repression [10].

Ankyrins, BTB/POZ and RCC1 domains are present in a wide range of proteins involved in different cellular processes, including gene expression regulation [8,12,13], cytoskeleton organization [14], and protein ubiquitination/degradation [9]. Thus, IBtkα could exert several regulatory roles through protein-protein interactions. Indeed, the involvement of IBtkα in tumor survival and cellular stress has been recently shown by (a) the viability loss of colorectal cancer cells DLD-1 K-Ras-positive cells by *IBTK* RNA interference [15]; (b) the increased IBtkα production in human bronchial epithelial cells exposed to the industrial pollutant Titanium dioxide [16], and in HeLa cells following the treatment with thapsigargin/tunicamycin, inducers of endoplasmic reticulum stress [17]. In addition, deletions of the *IBTK* gene have been reported in relapsed diffuse large B-cell lymphoma (DLBCL), the most common subtype of non-Hodgkin lymphoma in adults [18].

In this study, we have investigated the role of *IBTK* in the regulation of the human wide genome expression. In particular, we have performed High Throughput Deep RNA-Sequencing to analyze the transcriptome of epithelial (HeLa) and erythroleukemic (K562) cell lines, with or without *IBTK* RNA interference.

## 2. Results

### 2.1. Expression Profile of the IBTK Gene in Different Cellular Contexts

**Figure 1:**
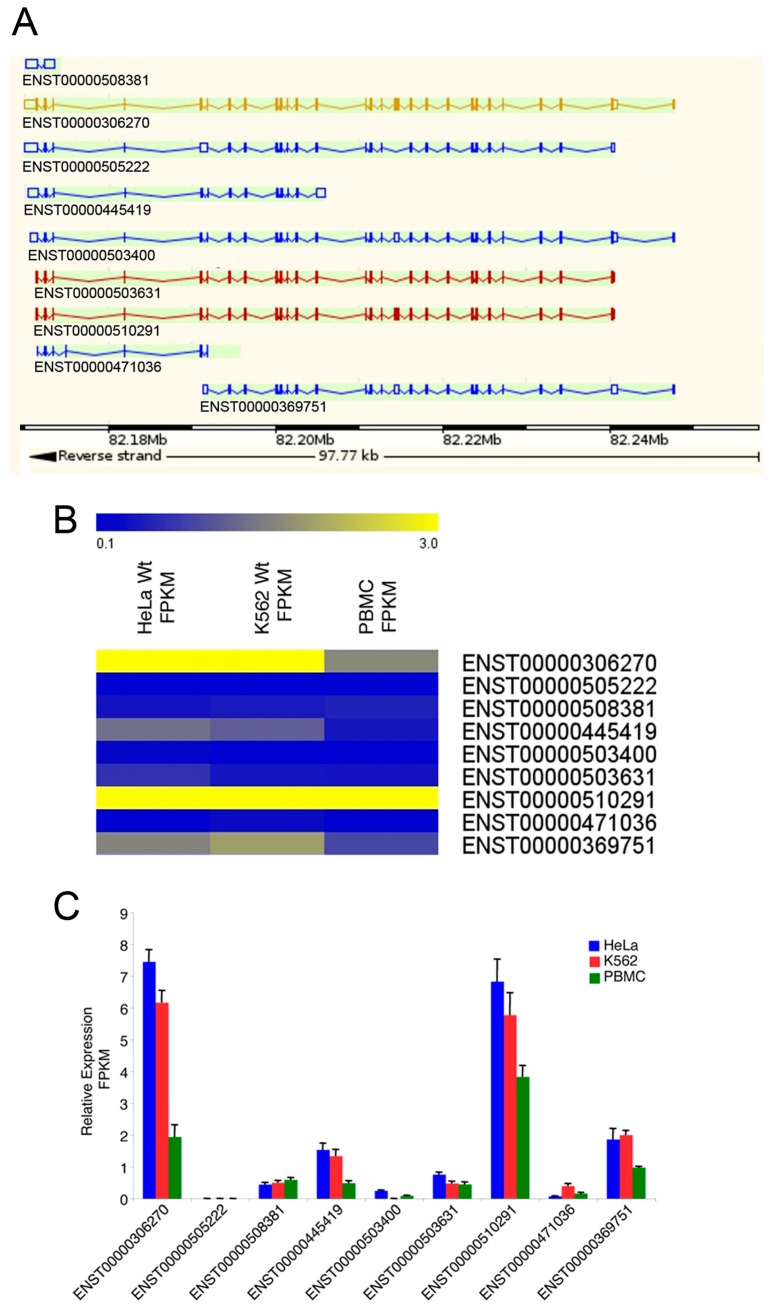
The *IBTK* gene expression profile in HeLa, K562, and PBMCs. (**A**) Schematic representation of *IBTK* transcripts, according to ENSEMBL genome browser; (**B**) Heatmap of *IBTK* transcript isoforms in HeLa, K562, and PBMCs; (**C**) Expression level of *IBTK* transcript isoforms in HeLa, K562, and PBMC was evaluated by measuring FPKM (Fragments per kilobase of exon per million fragments mapped). Values (mean ± SE, *n* = 3) are shown.

Based on ENSEMBL database (http://www.ensembl.org), the *IBTK* gene expresses different transcripts [1] (Figure 1A). To determine the expression of the *IBTK* transcripts in different cellular contexts, cDNA libraries were generated from HeLa, K562, and PBMCs, and subjected to High Throughput Deep RNA-Sequencing. The RNA-Seq short reads were mapped against the human genome (hg19 assembly). The expression levels of *IBTK* transcript isoforms were evaluated by measuring the Fragments Per Kilobase of Exon Per Million Fragments Mapped (FPKM). The most highly expressed transcripts in HeLa and K562 were ENST00000306270, corresponding to the canonical *IBTK*α mRNA [1], and ENST00000510291, corresponding to an alternatively spliced *IBTK*α isoform carrying a shorter 5′UTR and the deletion of the nucleotide sequence resulting in the loss of 15 amino acid residues at N-terminus of the IBtkα protein (1338 aa) (Figure 1A–C). In PBMCs, the transcript ENST00000510291 was slightly more expressed than ENST00000306270 (Figure 1A–C). The other *IBTK* isoforms, including *IBTK*β (ENST00000369751) and *IBTK*γ (ENST00000471036), were expressed at a minor level in all three cellular contexts. These results confirmed our previous observations in different human tissues and cell lines, indicating that the *IBTK*α isoform is the most abundant transcript as compared to *IBTK*β and *IBTK*γ [1]. Further, the occurrence of an alternative *IBTK*α isoform with a different 5′UTR suggests a possible regulatory mechanism of *IBTK* at the translational level.

### 2.2. Differential Gene Expression in IBTK-Silenced HeLa and K562 Cells

To analyze the effect of *IBTK* on the human transcriptome, we generated cDNA libraries from HeLa and K562 cells, which had been transduced with *IBTK*-shRNA or control-shRNA. The *IBTK* RNA interference was performed with retroviral particles that expressed the shRNA directed against the *IBTK* mRNA from nucleotide +1534 to +1552, encoding the amino acid 511 to 517 of the IBtkα protein. In HeLa and K562 cells, only the transcripts ENST00000306270 (canonical *IBTK*α) and ENST00000510291 (alternatively spliced *IBTK*α) were silenced with statistical significance, according to Student’s *t*-test (*p* < 0.05) (Supplementary Materials Figure S1).

**Figure 2:**
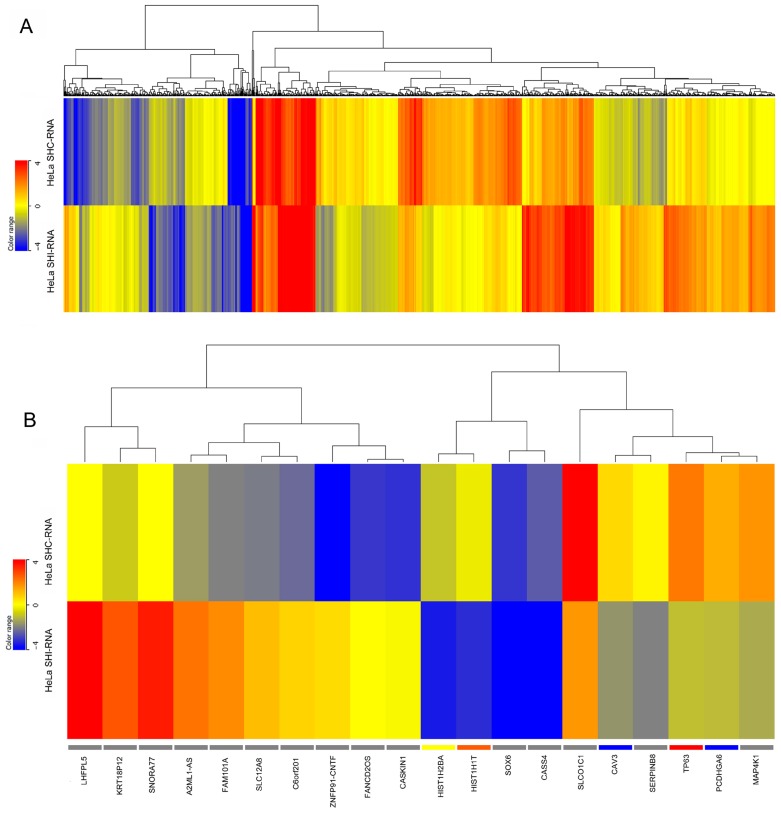
Heatmaps of gene expression in *IBTK*-shRNA- and control-shRNA-transduced HeLa. Total RNA was extracted from HeLa (1 × 10^6^ cells) transduced with viral particles expressing the *IBTK*-shRNA or control-shRNA, and subjected to RNA-Sequencing. (**A**) Heatmap of all differentially expressed genes in *IBTK*-shRNA- and control-shRNA-transduced HeLa; (**B**) Heatmap of the top 10 upregulated and downregulated differentially expressed genes in *IBTK*-shRNA- and control-shRNA-transduced HeLa.

**Figure 3:**
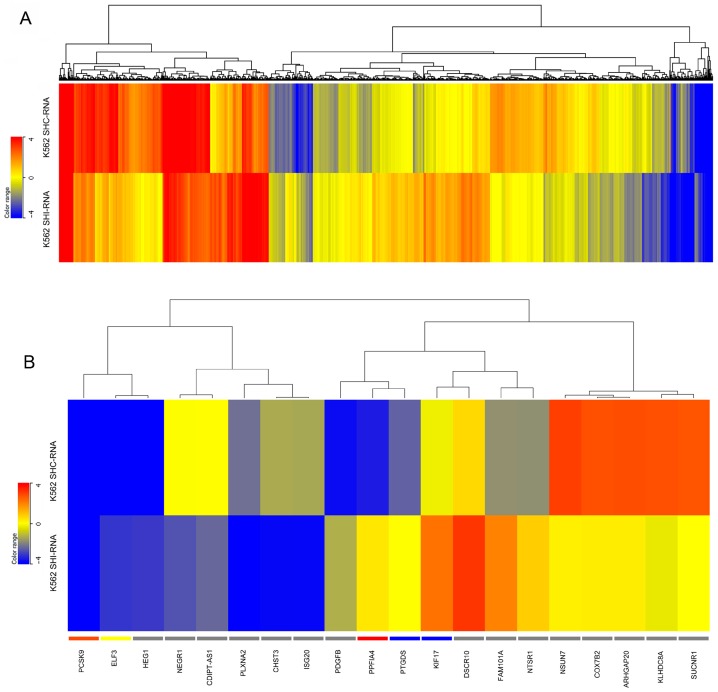
Heatmaps of gene expression in *IBTK*-shRNA- and control-shRNA-transduced K562. Total RNA was extracted from K562 (1 × 10^6^ cells) transduced with viral particles expressing *IBTK*-shRNA or control-shRNA, and subjected to RNA-Sequencing. (**A**) Heatmap of all differentially expressed genes in *IBTK*-shRNA- and control-shRNA-transduced K562; (**B**) Heatmap of the top 10 upregulated and downregulated differentially expressed genes in *IBTK*-shRNA- and control-shRNA-transduced K562.

By using fold change cutoff of ≥1.5 and *p* value ≤ 0.05, in HeLa cells, 1285 (2.03%) out of 63,128 mapped genes were differentially expressed in *IBTK*-shRNA as compared to control-shRNA, with 587 genes (45.7%) being upregulated and 698 genes (54.3%) downregulated (Figure 2). In K562 cells, 1959 (3.1%) out of 63,128 mapped genes were differentially expressed in *IBTK*-shRNA as compared to control-shRNA, being 1053 (53.7%) upregulated genes and 906 (46.3%) downregulated (Figure 3). The expression levels of differentially expressed genes in HeLa and K562, with or without *IBTK* RNA interference, are reported in Supplementary Materials Tables S1 and S2. Among the analyzed genes, a set of 137 genes (0.21%), including *IBTK*, was commonly deregulated by IBtk depletion in HeLa and K562 (Supplementary Materials Table S3). Of them, 33 genes were upregulated and 54 genes downregulated, while the remaining 50 genes were differently deregulated, being upregulated in one cell type and downregulated in the other one (Supplementary Materials Table S3). At the protein level, we verified the upregulation of Caveolin-3 and the downregulation of ULBP2 as consequence of *IBTK* silencing, which was consistent with the deregulation of these genes at the transcriptional level (Supplementary Materials Figure S2).

As the *IBTK*α transcript was the only isoform to be significantly silenced by *IBTK* RNA interference, the differential gene expression depending on *IBTK* and cellular context was likely due to the cell-specific interactions of the IBTKα protein with signalling molecules and transcription regulators. Further, the evidence of a small number of genes (0.14%) equally up- or downregulated by *IBTK* depletion in the two different cellular contexts indicated that the IBtkα protein likely modulated the activity of a few transcription regulators commonly expressed in HeLa and K562 cells.

Gene ontology analysis showed that the differentially expressed genes belong to different functional categories and are involved in different biological processes in the two cell lines. In HeLa cells, the genes deregulated by *IBTK* are mainly involved in nucleic acids metabolism, such as chromatin and nucleosomal organization and gene expression regulation (Supplementary Materials Figure S3), while in K562 cells, they are mainly involved in intra-cellular traffic, cell motility, and migration (Supplementary Materials Figure S4). This indicates that *IBTK* acts as transcriptional regulator of specific sets of genes, and this specificity depends on the cellular context.

### 2.3. IBTK Affects Splicing Events in HeLa and K562 Cells

The analysis of RNA-seq data is extremely useful in characterizing alternative splicing events [19]. Thus, we asked whether *IBTK* could affect the splicing process in HeLa and K562 cells. We used Multivariate Analysis of Transcript Splicing (MATS) (http://rnaseq-mats.sourceforge.net/) as a computational tool to detect differential alternative splicing events from RNA-Seq data [20,21]. To this end, we scanned all transcripts encoded by the whole genome for splicing patterns occurring in *IBTK*-shRNA transduced HeLa and K562 cells, as compared to control-shRNA.

**Figure 4:**
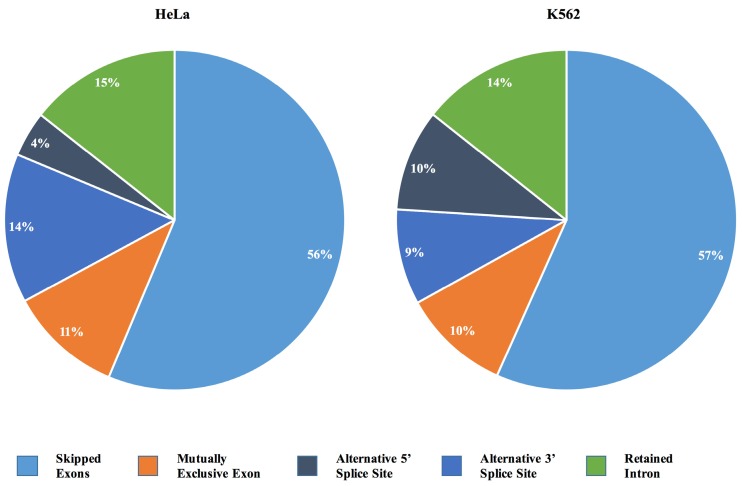
Pie chart of alternative splicing events in *IBTK*-shRNA- or control-shRNA-transduced HeLa and K562. Alternative splicing events were analyzed by MATS. Only events showing *p* ≤ 0.05, FDR ≤ 0.05 and a minimum inclusion level difference ≥0.1 were considered.

Based on cutoff of *p* ≤ 0.05 and a minimum inclusion level difference ≥0.1, *IBTK* depletion caused alternative splicing events in both HeLa and K562 cells (Supplementary Materials Tables S4 and S5). In particular, 1481 alternative splicing events occurred in HeLa and 1660 in K562, with similar frequencies of the different categories of splicing events, including alternative 3′- and 5′-splicing, mutually exclusive exons, retained intron and skipped exons (Figure 4). Among the alternative splicing events, exon skipping was the most common one (834 events in HeLa and 941 events in K562). MATS analysis also indicated a higher frequency of retained intron events (213 in HeLa and 238 in K562), mutually exclusive exons (160 events in HeLa and 170 events in K562), and a lower frequency of alternative 5′-splicing events (64 in HeLa and 151 in K562) and alternative 3′ splicing (210 events in HeLa and 160 in K562) (Figure 4). More specifically, in both HeLa and K562, we found equally alternative splicing events, including 50 at 3′, 12 at 5′, 23 mutually exclusive exons, 44 retained introns, and 164 skipped exons.

**Figure 5:**
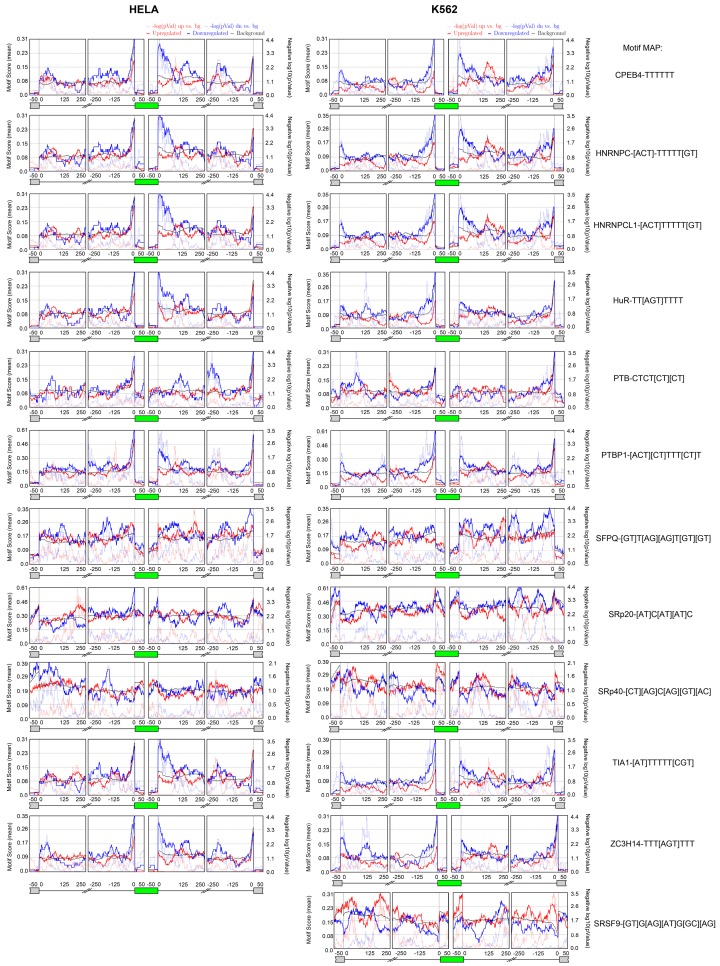
Identification of RBPs (RNA Binding Proteins) and relative conserved motifs within the *IBTK*-dependent alternatively spliced genes. Alternative splicing events were analyzed by MATS; conserved motifs within alternatively spliced genes were analyzed by rMAPS. Only events showing *p* ≤ 0.05, FDR ≤ 0.05 and a minimum inclusion level difference ≥0.1 were considered.

Then, we asked the question of which splicing factors could regulate the alternative splicing events, and whether a specific consensus is conserved within the alternatively spliced genes upon *IBTK* depletion. To this end, we analyzed the MATS outputs using the bioinformatics software rMAPS (http://rmaps.cecsresearch.org/) [22], which systematically generates RNA-maps for the identification of consensus sequences of RNA-binding proteins (RBPs) with position-dependent functions. In particular, the rMAPS program is extremely useful for the computational detection of binding sites around differential alternative splicing events for over 100 of known RBPs. The rMAPS-based analysis, using the default parameters, identified similar conserved sequence motifs within the *IBTK*-dependent alternatively spliced genes in the two cellular contexts (Figure 5). In particular, in both HeLa and K562, 11 potential RNA binding proteins (CPEB4, HNRNPC, HNRNPCL1, HuR, PTB, PTBP1, SFPQ, SRp20, SRp40, TIA1, ZC3H14) with the relative conserved consensus motifs were identified in the *IBTK*-induced alternative spliced events, while one additional RNA binding protein was specific for K562 (SRSF9) (Figure 5).

## 3. Discussion

The transcriptome analysis here performed by high throughput deep RNA-sequencing provides new insights into the modulation of the human wide genome expression in dependence of *IBTK*. The experimental approach was to interfere the expression of *IBTKα*, the major transcript isoform of the *IBTK* gene, which encodes the *IBTK*α protein, a component of Cul3-dependent E3 Ligase [10]. Since IBtkα contains the protein-protein interaction domains ankyrin repeats, RCC1 and BTB/POZ, we do not exclude that the depletion of this protein could result in the loss of interaction with several cellular targets, which have yet to be characterized. To date, the only substrate recognized to be ubiquitinated through CRL3^IBTK^ is Pdcd4 [10]; however, the characterization of the full interactome of IBtkα could reserve expanded regulatory roles of this protein.

This study has provided the preliminary evidence of the involvement of IBtkα in the regulation of gene expression in HeLa and K562 cell lines. Given the pleiotropic activity of IBtkα as substrate receptor of Cul3 ubiquitin ligase and negative regulator of Btk-dependent signalling in B-cells, it is quite difficult at this time to determine the molecular mechanisms of IBtkα in wide genome expression regulation. We are currently running an extensive analysis of the IBtkα interactome in different cellular contexts with the aim to identify relevant cellular partners that could be regulated by IBtkα, which could explain the pleiotropic effects of this protein, including gene expression regulation. Indeed, the modulation of gene expression could be achieved through direct interaction of IBtkα with transcriptional regulators, being substrates of ubiquitination coupled to proteasomal degradation. In this regard, IBtkα could promote NF-κB-dependent transcription through ubiquitination coupled to proteasomal degradation of Pdcd4, an inhibitor of p65/NF-κB [23]. Furthermore, IBTKα could act in cell-type specific signalling targeting other intracellular molecules. For example, the shorter isoform IBtkγ, which shares the homology amino acid sequence with the carboxy-terminal of IBtkα, inhibits Btk through physical interaction, and indirectly inhibits the signalling of NF-κB activation dependent on Btk [2,4].

The data shown here demonstrate that IBtkα also affects RNA splicing in HeLa and K562, which could represent an additional mechanism of regulation of cellular functions. By computational analysis, we have identified a set of RNA-binding proteins with the relative sequence motifs that commonly occur within the *IBTK*-dependent alternatively spliced genes in HeLa e K562, suggesting that a few regulatory post-transcriptional proteins are a common target of IBtkα in both cellular contexts. In this regard, it is worthwhile to remember that changes in splicing patterns affect biological processes by many different mechanisms, including gain-of-function or functional switches, altered cellular localization, dominant negative effect, or changes in protein/mRNA stability [24].

The amplitude of cellular processes that can be affected by IBtkα through modulation of gene expression and alternative splicing suggests an expanded role of IBtk in cell biology, which is mostly depending on cellular context.

## 4. Experimental Sections

### 4.1. Cells

HeLa, K562, and HEK293T cell lines were purchased from the American Type Culture Collection, Manassas, VA, USA. HeLa and HEK293T were cultured in Dulbecco’s modified Eagle’s medium supplemented with 10% heat-inactivated foetal calf serum and 2 mM l-glutamine (Lonza Cologne AG, Cologne, Germany); K562 was cultured in RPMI1640 (Lonza Cologne AG, Cologne, Germany) supplemented with 10% heat-inactivated foetal calf serum and 2 mM l-glutamine. Human peripheral blood mononuclear cells (PBMCs) were derived from buffy coats of three healthy donors and isolated by Ficoll Paque gradient (GE Healthcare Europe, Munich, Germany), as previously described [25,26,27]. Briefly, blood samples were diluted 1:1 in PBS and stratified on Ficoll solution with a 3:1 (*v*/*v*) ratio. After 30 min centrifugation at 1200× *g*, PBMCs were recovered and re-suspended in RPMI-1640 medium supplemented with 10% foetal calf serum.

### 4.2. Plasmids and Lentiviral Infections

The plasmids pCMV-dR8.91 and pCMV-VSVG were purchased from AddGene (OneKendall, Cambridge, MA, USA). The lentiviral constructs expressing the *IBTK*-shRNA (TRCN0000082575) or control shRNA (SHC002) were purchased from MISSION (SigmaAldrich, St. Louis, MO, USA). The *IBTK*-shRNA targets the nucleotide sequence from +1534 to +1552 nucleotide of the *IBTK*α transcript (Ensemble Reference Sequence: ENST00000306270). Lentiviral stocks were produced by transfection of HEK293Tcells, as previously described [28]. Briefly, HEK293Tcells (1 × 10^6^) were transfected with pCMV-dR8.91 (5 µg) and pCMV-VSVG (5 µg) together with *IBTK*-shRNA (10 µg) or control-shRNA (10 µg); 48-h post-transfection, cell supernatant was collected. Enzyme-linked immunosorbent assay (ELISA) using anti-p24 antibody measured virions concentration [27]. HeLa or K562 cells (1 × 10^6^) were infected with viral stocks (500 ng of p24) by spinoculation, as previously described. To select cell clones, stably expressing *IBTK*-shRNA or control-shRNA, puromycin (1 µg/mL) was added to cell cultures 48 h after infection.

### 4.3. Cells Extracts and Western Blotting

Protein extracts were obtained as previously described [25,29]. Briefly, HeLa and K562 cells were harvested, washed twice with PBS 1X and lysed in RIPA buffer, containing 1% NP-40, 10 mM Tris-HCl, 150 mM NaCl, and 1 mM EDTA, supplemented with a protease inhibitor cocktail (cOmplete-mini EDTA-free tablets—Roche) on ice for 20 min. Lysates were clarified by centrifugation at 14,000× *g*, 4 °C for 10 min. Proteins (30µg) were resolved on Novex NuPage 12-4% (ThermoFisher Scientific, Carlsbad, CA, USA) and transferred to polyvinylidene difluoride membrane (Millipore, Bedford, MA, USA) and incubated with primary antibodies (1:1000) followed by incubation with horseradish-peroxidase-linked mouse or rabbit IgG (1:2000) (GE Healthcare Amersham, Little Chalfont, UK) in PBS containing 5% non-fat dry milk (Bio-Rad Laboratories, Hercules, CA, USA). Proteins were detected by chemiluminescence using the ECL System (GE Healthcare, Amersham, UK). Primary antibodies were purchased from Sigma-Aldrich (anti-γ-Tubulin and anti-Caveolin-3), Bethyl Laboratories (anti-IBtkα), and Abcam (anti-ULBP2).

### 4.4. RNA Sequencing

RNA extraction from the pooled PBMCs samples, and from *IBTK*-shRNA or control-shRNA transduced HeLa and K562 was performed as previously described [30]. RNA concentration was determined with a ND-1000 spectrophotometer (NanoDrop, Wilmington, DE, USA), and its quality was assessed with an Agilent 2100 Bioanalyzer using Agilent RNA 6000 nano kit (Agilent Technologies, Waldbronn, Germany). For library preparation, a starting amount of 5 μg RNA per sample was used; rRNA was depleted using the Ribo-Zero rRNA Removal Kit (Human/Mouse/Rat; Epicenter, Madison, WI, USA). The purified RNA was used for indexed libraries preparation with TruSeq RNA Sample Prep Kit (Illumina, San Diego, CA, USA), according to the manufacturer’s instructions. Libraries were quantified using the Agilent 2100 Bioanalyzer (Agilent Technologies) and pooled to obtain equimolar amounts of each index-tagged sample, with final concentration of the pooled samples of 2 nM. The pooled samples were subject to cluster generation and sequencing using an Illumina GaIIx (Illumina) in a 2 × 72 paired-end format at a final concentration of 8 pmol. Data analysis was performed as described [31], with minor variations. In brief, the raw sequence files generated (.fastq files) underwent quality control analysis using FastQC (http://www.bioinformatics.babraham.ac.uk/projects/fastqc/) and the quality checked reads were then aligned to the human genome (hg19 assembly) using TopHat version 2.0.10, using standard parameters. The expression value of each mRNA was normalized to Fragments Per Kilobase of exon model per Million of sequenced reads (FPKM) as computed by Cufflink v2.1.1 [32]. A given RNA was considered expressed when detected by ≥10 reads. Differentially expressed RNAs were identified using DESeq version 1.14.0 [33]. Gene annotation was obtained for all known genes in the human genome, as provided by Ensemble (GRCh37) (https://support.illumina.com/sequencing/sequencing_software/igenome.ilmn). Using the reads mapped to the genome, the number of reads mapping to each transcript was calculated with HTSeq-count (http://www-huber.embl.de/users/anders/HTSeq/doc/overview.html). These raw read counts were then used as input to DESeq for calculation of normalized signal for each transcript in the sample, and differential expression was reported as Fold Change (FC) along with associated adjusted *p*-values (computed according to Benjamini-Hochberg). Heatmaps were generated with Multiexperiment Viewer 4.9 (TM4). Gene ontology analysis of differentially expressed genes was performed with GORILLA (Gene Ontology enRIchment anaLysis and visuaLizAtion tool) software (http://cbl-gorilla.cs.technion.ac.il/). RNAseq date have been deposited to SRA (Sequence Read Archive) (http://www.ncbi.nlm.nih.gov/sra) with Accession Number SRP079879.

### 4.5. Ethics Statement

Human blood samples were obtained from healthy donors after informed consent in accordance with the principles expressed in the Declaration of Helsinki. The protocol of the study was approved by the Ethics Committee of University of Catanzaro “Magna Graecia”, Catanzaro, Italy in strict accordance with Italian Ministry of Health (Permit Number: 0008613-P) and directives of European Community Council C.E. 5 December 2002, C.E. 2 December 2004, and C.E. 3 March 2005.

## Supplementary Materials

Supplementary materials can be found at www.mdpi.com/1422-0067/17/11/1848/s1.
