# Supplementary material for: IBTK Differently Modulates Gene Expression and RNA Splicing in HeLa and K562 Cells
Source: Int J Mol Sci. 2016 Nov 7;17(11):1848. doi: 10.3390/ijms17111848 (PMC5133848; doi:10.3390/ijms17111848)
Supplement: Supplementary file 1 [file ijms-17-01848-s001.zip › ijms-144583-Supplementary Materials/ijms-144583-Supple-Figure S1-S4.pdf]

Supplementary Materials: IBTK Differently Modulates Gene Expression and RNA Splicing in HeLa and K562 Cells

Giuseppe Fiume, Annarita Scialdone, Francesca Rizzo, Maria Rosaria De Filippo, Carmelo Laudanna, Francesco Albano, Gaetanina Golino, Eleonora Vecchio, Marilena Pontoriero, Selena Mimmi, Simona Ceglia, Antonio Pisano, Enrico Iaccino, Camillo Palmieri, Sergio Paduano, Giuseppe Viglietto, Alessandro Weisz, Giuseppe Scala and Ileana Quinto

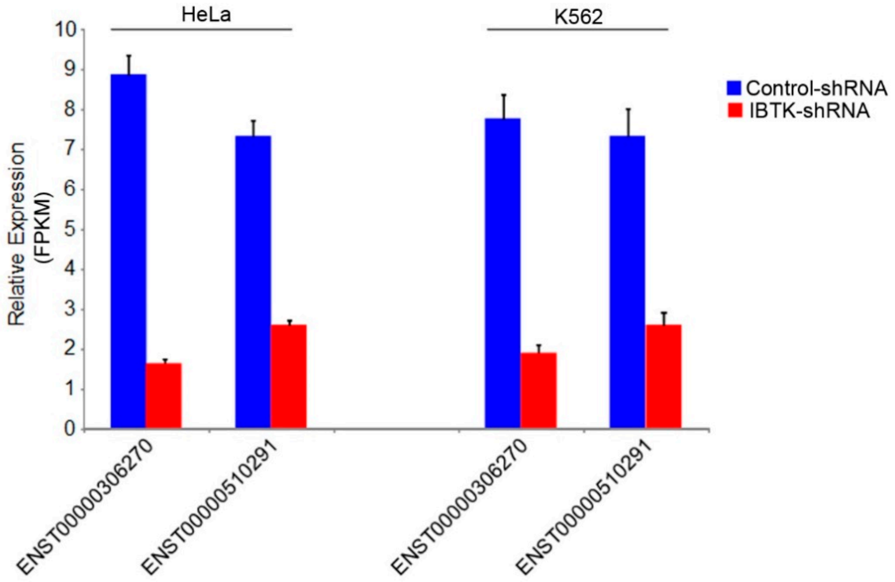

Figure S1. *IBTK* transcripts expression in *IBTK*-shRNA- and control-shRNA-transduced HeLa and K562. Expression level of *IBTK* transcript isoforms in *IBTK*-shRNA- and control-shRNA-transduced HeLa and K562 was evaluated by measuring FPKM. Values (mean ± SE, $n = 3$) are shown.

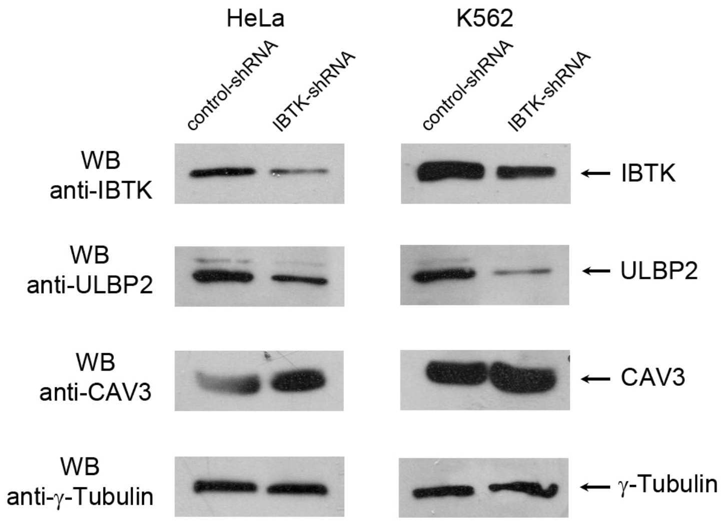

Figure S2. Altered expression of ULBP2 and Caveolin-3 at protein level upon *IBTK* depletion in HeLa and K562. Western blotting analysis of *IBTKα*, ULBP2, CAV3 and γ-Tubulin in total protein extracts (30 μg) of *IBTK*-shRNA- and control-shRNA-transduced HeLa and K562.

Gene Ontology of differentially expressed genes
by *IBTK* depletion in HeLa

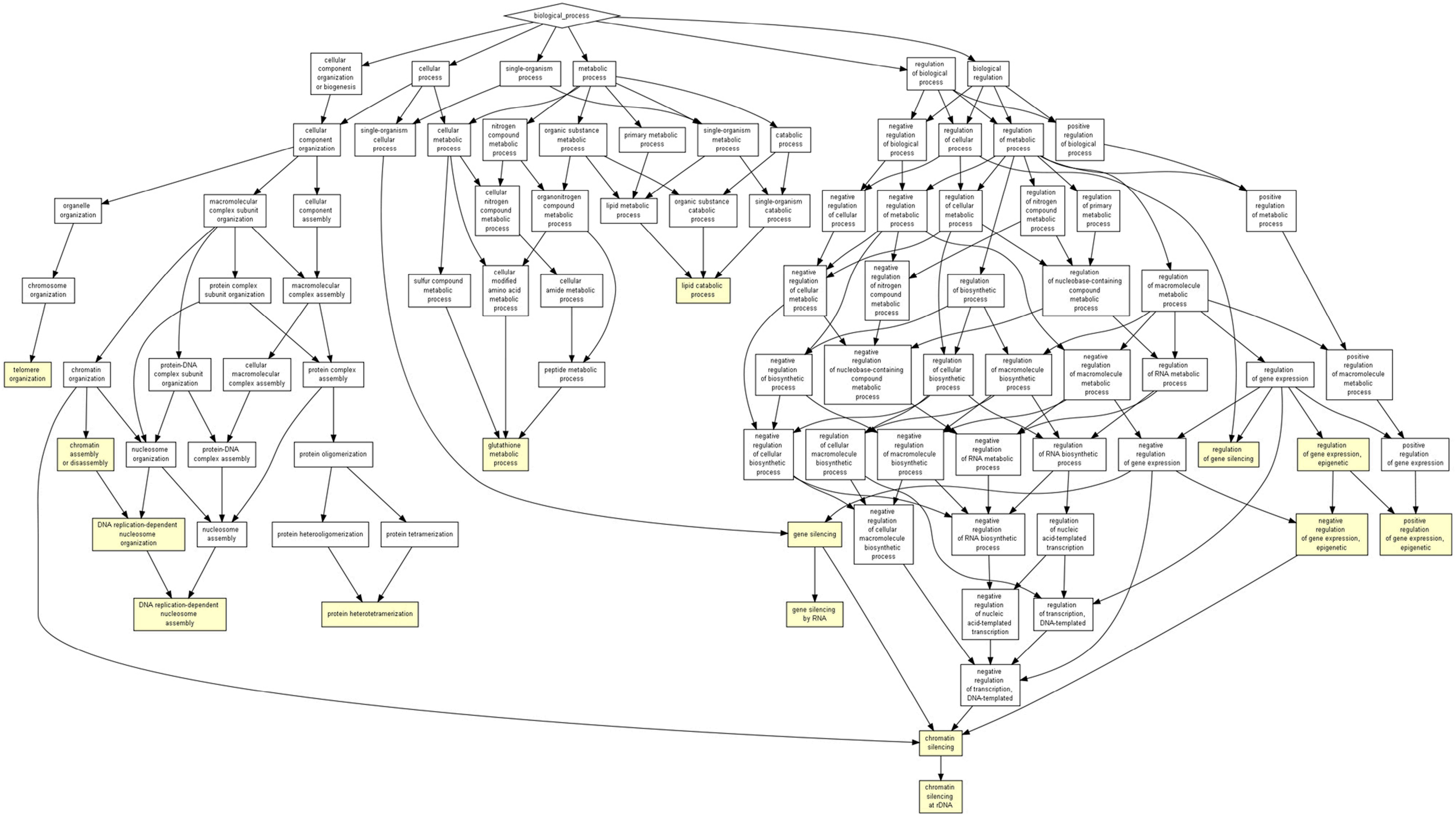

Figure S3. Gene Ontology analysis of genes differentially expressed in HeLa cells following *IBTK* depletion.

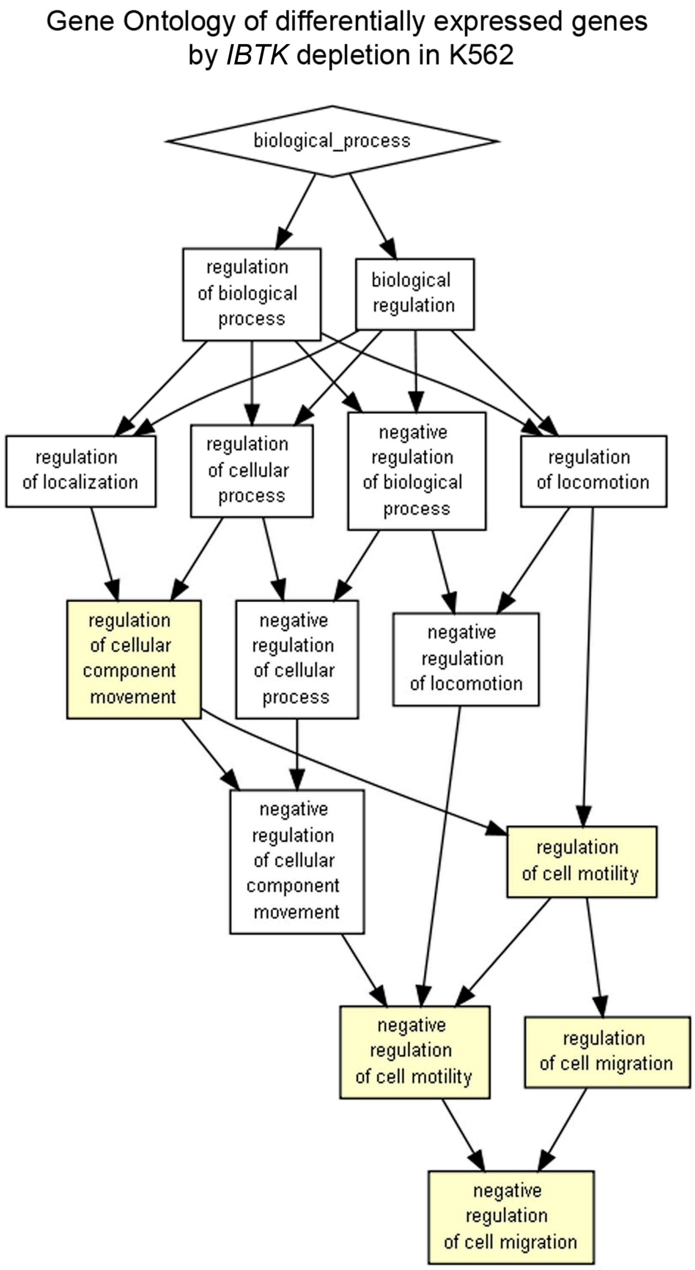

Figure S4. Gene Ontology analysis of genes that are differentially expressed in K562 cells upon *IBTK* depletion.

Table S1. List of differentially expressed genes by *IBTK* depletion in HeLa cells.
Table S2. List of differentially expressed genes by *IBTK* depletion in K562 cells.
Table S3. List of commonly deregulated by *IBTK* depletion in HeLa and K562 cells.
Table S4. Alternative splicing events upon *IBTK* depletion in HeLa cells.
Table S5. Alternative splicing events upon *IBTK* depletion in K562 cells.
